# Hydrogen gas (H_2_) delivered by intraperitoneal injection alleviated methionine- and choline-deficient diet-induced metabolic dysfunction–associated steatotic liver disease in mice via inhibiting GSDMD- and GSDME-mediated pyroptosis

**DOI:** 10.3389/fphar.2025.1575106

**Published:** 2025-08-08

**Authors:** Yun Chen, Kangrong Wang, Wenhai Guo, Chengqin Lu, Wenting Suo, Qiuling Li, Yao Deng, Xinling Chen, Min Dai, Xiaodong Zhang, Jiean Xu, Wen Su, Shuangling Yang, Hongzhi Yang, Fuman Yan, Haimei Liu, Yaxing Zhang

**Affiliations:** ^1^ Department of Physiology, School of Basic Medical Sciences, Guangzhou University of Chinese Medicine, Guangzhou, Guangdong, China; ^2^ Research Centre of Basic Integrative Medicine, School of Basic Medical Sciences, Guangzhou University of Chinese Medicine, Guangzhou, Guangdong, China; ^3^ Department of Traditional Chinese Medicine, The Third Affiliated Hospital, Sun Yat-sen University, Guangzhou, Guangdong, China; ^4^ Institute of Integrated Traditional Chinese and Western Medicine, Sun Yat-sen University, Guangzhou, Guangdong, China; ^5^ Department of Allergy and Immunology, The Third Affiliated Hospital, Sun Yat-sen University, Guangzhou, Guangdong, China; ^6^ School of Health Sciences, Guangzhou Xinhua University, Guangzhou, Guangdong, China

**Keywords:** intraperitoneal injection, MCD, TLR4, NLRP3, GSDMD, GSDME, pyroptosis

## Abstract

**Background:**

Hydrogen gas (H_2_), which is the lightest and diffusible gas molecule, has strong abilities to alleviate excessive oxidative stress, inflammation, and apoptosis. Inhalation of H_2_ is beneficial for preventing the damage of the lung, heart, brain, liver, kidneys, and many other organs. However, the effect of intraperitoneal injection of H_2_ on metabolic dysfunction–associated steatotic liver disease (MASLD) is unclear.

**Objective:**

The aim of this study is to investigate whether intraperitoneal injection of H_2_ can improve MASLD, and if so, what are the key innate immune mechanisms involved?

**Methods:**

The MASLD mouse model was established by feeding a methionine- and choline-deficient (MCD) diet for 3 weeks. H_2_ was daily given by intraperitoneal injection since the eighth day of MCD diet feeding, and lasted for 2 weeks. Serum levels of alanine aminotransferase (ALT) and aspartate aminotransferase (AST) were examined to evaluate liver injury. Hematoxylin and eosin (H&E) staining, Oil Red O staining, qPCR analysis of hepatic lipid metabolism genes, and detection of hepatic triglyceride (TG) levels were performed to evaluate hepatic steatosis. Masson trichrome staining and Collagen-I and Collagen-III protein levels were used to evaluate liver fibrosis. The liver 3-nitrotyrosine (3-NT) was detected by immunoblotting and immunofluorescence, and the levels of malondialdehyde (MDA) and reduced glutathione (GSH) were measured using kits to evaluate redox homeostasis. The activation of TLR4-mediated innate immune signaling and pyroptosis were tested by immunoblotting and immunofluorescence. Moreover, hepatic protective effect and anti-pyroptosis effect of H_2_ were further confirmed by H_2_-rich DMEM-treated HepG2 cells *in vitro*.

**Results:**

Supplementing with H_2_ by intraperitoneal injection protected MCD diet-fed mice against hepatic steatosis and fibrosis by down-regulating *de novo* lipogenesis and fatty acid uptake genes, as well as hepatic Collagen-Ⅰ and Collagen-Ⅲ protein levels, while up-regulating lipid export genes. Mechanistically, H_2_ modulated hepatic redox homeostasis by suppressing 3-NT and MDA levels, while increasing the reduced GSH levels. Subsequently, reactive oxygen species (ROS)-related innate immune signaling, including the expression of TLR4, and the activation of NF-κB, ERK1/2, p38 MAPK, and JNK in the liver, were all inhibited by H_2_ treatment. These further contributed to inhibiting the expression of TNF-α, IL-1β, and IL-18 in the liver. The maturation of IL-1β and IL-18, the full-length of the classical pyroptosis trigger GSDMD, and the cleavage of GSDMD processed by Caspase-1 in NLRP3 inflammasome (including NLRP3, ASC, Caspase-1) were all blocked by H_2_. In addition, H_2_ decreased both the full-length and cleaved forms of Caspase-11, Caspase-8, Caspase-3 and GSDME, and thus inhibiting the non-canonical pyroptosis signaling in the liver of MASLD mice. The anti-pyroptosis effects of H_2_ *in vitro* were further confirmed by the reduced expression of inflammatory cytokines, the decreased full-length and cleaved forms of GSDMD and GSDME, and the reduced number of HepG2 cells with pyroptotic morphology.

**Conclusion:**

H_2_ is an anti-pyroptosis gas molecule, intraperitoneal injection of H_2_ is a novel therapeutic strategy for MASLD that deserves further investigation.

## 1 Introduction

Metabolic dysfunction–associated steatotic liver disease (MASLD, formerly known as non-alcoholic fatty liver disease (NAFLD)) is a dynamic chronic non-communicable liver disease, which displays hepatic steatosis with one or more cardiometabolic risk factors in the absence of other causes of hepatic steatosis, such as drug-induced or alcohol-related steatosis (Sarkar and Kushner, 2025). MASLD includes a broad spectrum of progressive steatotic liver conditions, ranging from isolated hepatic steatosis to metabolic dysfunction-associated steatohepatitis (MASH) with varying amounts of liver fibrosis, and 10%–20% of the patients still progress to cirrhosis and its complications—hepatocellular carcinoma (HCC) and end-stage liver disease (Chen et al., 2021; Mallet et al., 2024; Miao et al., 2024). The global prevalence of MASLD was estimated to be 30.2% (Amini-Salehi et al., 2024). Resmetirom, a liver-selective and thyroid hormone receptor B (THRB, the predominant thyroid hormone receptor isoform in the liver)-selective thyromimetic, is the only one and first FDA-approved drug for MASH (Sinha et al., 2024). Therefore, it is still urgent to find other effective therapeutic drugs for MASLD.

Oxidative stress, inflammation, insulin resistance, and cell death are implicated in the pathogenesis of MASLD (Zhang et al., 2020c). Hydrogen gas (H_2_), the lightest gas molecule in nature, has antioxidant, anti-inflammatory, and anti-apoptotic effects (Tan et al., 2019). Inhalation of H_2_, drinking H_2_-rich water and intraperitoneal injection of H_2_-rich saline can alleviate many liver diseases, such as ischemia/reperfusion (I/R) injury and MASLD (Sun et al., 2011; Zhai et al., 2017; Ge et al., 2019; Liang et al., 2023; Liu et al., 2023). Our group and others have shown that intraperitoneal injection of H_2_ can alleviate acute alcoholic liver injury and lipopolysaccharide (LPS)-induced cardiac dysfunction in mice (Tan et al., 2019; Zhang et al., 2021a; Xu et al., 2024), and display the neuroprotection in rabbits with cardiac arrest (Huang et al., 2013). However, the therapeutic effect of intraperitoneal injection of H_2_ on MASLD are unknown, if it has a liver protective effect, what is the key mechanism?

## 2 Materials and methods

### 2.1 H_2_ and H_2_-rich medium

**FIGURE 1:**
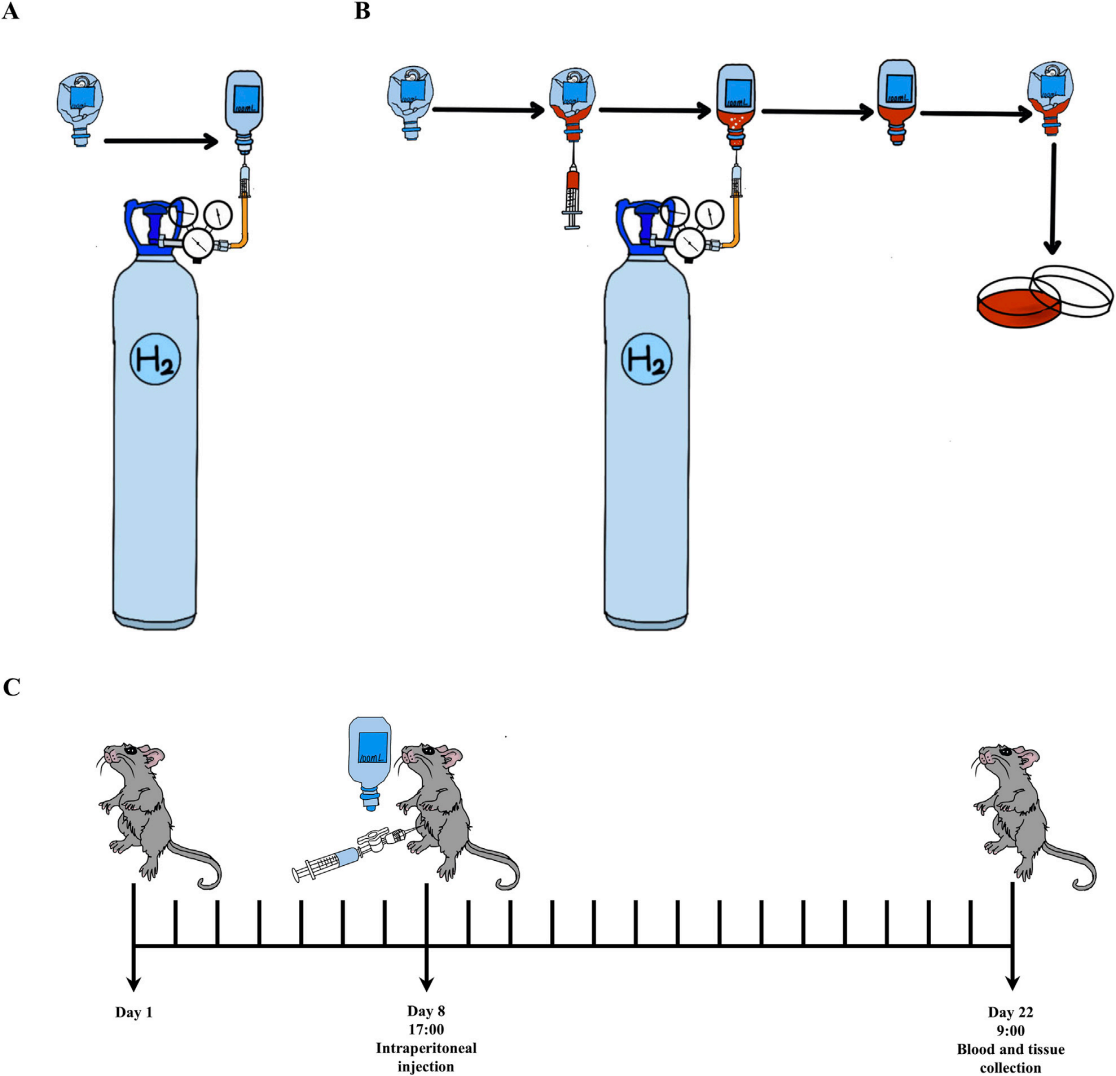
The methods of H_2_ usage and experimental process. **(A)** Open the pressure reducing valve with a small release pressure, exhaust the air in the plastic hose connected to the pressure reducing valve. Insert the syringe needle into the evacuated plastic infusion bottle (100 mL), then further loosen the pressure reducing valve and introduce H_2_ into the infusion bottle until there is no dead volume. **(B)** H_2_-rich medium was prepared by introducing H_2_ into the infusion bottle containing 10 mL DMEM until there is no dead volume, then, it was stored in 4°C for 6 h before used. **(C)** The experimental procedure is as follows: animals in H_2_ treatment group are intraperitoneally injected with H_2_ once a day at 5:00 p.m. since the eighth day. On day 22nd at 9:00 a.m., blood and liver samples are collected. In detail, there is a two-way valve connected between the injector and needle for injecting H_2_. Open the two-way valve, extract the required volume of H_2_ into the syringe, and immediately close the two-way valve. After removing the needle from the infusion bottle, immediately insert the needle into the abdominal cavity of the animals, open the two-way valve to complete the intraperitoneal injection.

H_2_ used for animals was daily prepared by injecting H_2_ (Cat#73405157, Dalian Special Gases Co., Ltd., Dalian, China; >99.999%) into a 100 mL vacuumed infusion bottle (Guangdong Kelun Pharmaceutical Co., Ltd., Meizhou, China). H_2_-rich Dulbecco’s modified Eagle’s medium (DMEM) for cell culture was prepared as we previously described. All these were shown in Figures 1A,B.

### 2.2 Animal model of metabolic dysfunction-associated steatotic liver disease and H_2_ treatment

The male C57BL/6 mice were brought from Guangdong Medical Laboratory Animal Center (Foshan, China). They were housed in an SPF animal facility with a 12-h light/dark cycle and *ad libitum* access to diet and water. All experimental procedures of animals were approved by the Institutional Animal Care and Use Committee (IACUC) of Guangzhou University of Chinese Medicine as a sub project of the “Building a Comprehensive Prevention and Treatment System for Cardiometabolic Diseases (3-1)” (Approval No. 2023053006).

The MASLD mouse model was established by feeding a MCD diet for 3 weeks (Henao-Mejia et al., 2012; Valdecantos et al., 2017; Figure 1C). Two doses of H_2_ were used in this study (Xu et al., 2024). C57BL/6 mice (26–28 g) were randomly divided into four groups, including Control group, MCD group, MCD + H_2_ (Low, L) group, and MCD + H_2_ (High, H) group. The animals in the corresponding group were fed a mixed diet for 3 days, as 50% standard chow plus 50% MCD Control diet (Cat#MD12051, Medicience Ltd., Yangzhou, China) for Control group, and 50% standard chow plus 50% MCD diet (Cat#MD12052, Medicience Ltd., Yangzhou, China) for the other three groups, and subsequently, mice in the corresponding group were fed a MCD Control diet or a MCD diet for another 18 days. On day eighth, mice were daily given H_2_ by intraperitoneal injection at the doses of 0.5 mL/100 g for MCD + H_2_ (L) group, and 1.0 mL/100 g for MCD + H_2_ (H) group. On day 22nd, mice were deeply anesthetized before blood collection from the orbital sinus, and then, euthanized via cervical dislocation (Mackowiak et al., 2022). The liver samples were frozen in −80°C or put in 4% Paraformaldehyde Fix Solution (Cat#G1101, Servicebio, Wuhan, China) for further analysis.

### 2.3 Sodium oleate-induced lipid accumulation in HepG2 cells and treatment by H_2_-rich medium

HepG2 cells, which were generously provided by Prof. Peng Zhang (School of Basic Medical Sciences, Wuhan University, China), were cultured in DMEM containing 10% fetal bovine serum (FBS) and 1% penicillin-streptomycin in a cell culture incubator with 5% CO_2_ at 37°C. Oleic acid (OA)-induced lipid accumulation HepG2 cell model was established by sodium oleate (Cat#S6130, Solarbio, Beijing, China) (Chen et al., 2020; Liu et al., 2020; Wang et al., 2022). HepG2 cells were divided into four groups: Control group, OA group, OA+H_2_ group, and H_2_ group. H_2_-rich DMEM with 1% FBS was added to the OA+H_2_ and H_2_ groups, and the Control group and OA group were treated with the same volume of H_2_-free DMEM with 1% FBS. HepG2 cells in the OA group and OA+H_2_ group were treated with sodium oleate (0.25 mM) for 18 h after 30 min of H_2_ treatment.

### 2.4 Serum ALT and AST, and hepatic MDA, reduced GSH and TG analysis

Serum levels of alanine aminotransferase (ALT) and aspartate aminotransferase (AST) were examined by an automatic blood chemistry analyzer in Department of Clinical Laboratory from The Third Affiliated Hospital of Sun Yat-sen University (Xu et al., 2024). Hepatic malondialdehyde (MDA), reduced glutathione (GSH), and triglycerides (TG) levels were examined by the commercialized reagent kits (Cat#A003-1-2, A006-2-1, and A110-1-1, Nanjing Jiancheng Bioengineering Institute, Nanjing, China).

### 2.5 Histopathological analysis

The fixed liver tissues by 4% paraformaldehyde fixed solution for over 24 h were embedded in paraffin and sliced into 4 μm sections. Then, Masson’s trichrome (Cat#G1006-100ML, Servicebio, Wuhan, China) staining, and Hematoxylin (Cat#BA4097-500ML, BaSo, Zhuhai, China) and eosin (Cat#BA4099-500ML, BaSo, Zhuhai, China) (H&E) staining was performed according to the standard procedures. In H&E-stained pathological sections, hepatic steatosis was scored and the severity was graded based on the percentage of affected total area, into the following categories: 0 (<5%), 1 (5%–33%), 2 (>33–66%), and 3 (>66%) (Kleiner et al., 2005; Liang et al., 2014; Zhang et al., 2020c). The H&E image analysis was performed by Chengqin Lu and Yun Chen, and a double-blind design was implemented to minimize bias. HepG2 cells were fixed with 4% paraformaldehyde, and the frozen liver sections (10 μm) were prepared in Tissue-Tek^®^ optimum cutting temperature (O.C.T.) compound (Cat#4583, Sakura, Japan), then, they stained with Oil Red O (Cat#G1015-100ML, Servicebio, Wuhan, China). Hepatic lipid accumulation by Oil Red O staining in the liver tissues were quantified as previously described (Mehlem et al., 2013).

### 2.6 Quantitative PCR (qPCR) analysis

Total RNA was extracted from the fresh liver tissues by EZ-press RNA Purification Kit (Cat#B0004DP, EZBiocience, Roseville, CA, United States), Color Reverse Transcription Kit (Cat#A0010CGQ, EZBiocience, Roseville, CA, United States) was used to convert mRNA to cDNA, and 2 * Color SYBR Green qPCR Master Mix (Cat#A0012-R2, EZBiocience, Roseville, CA, United States) was used for qPCR under the standard procedure. The target genes levels were normalized to glyceraldehyde-3-phosphate dehydrogenase (*Gapdh*) by 2^−△△ct^. The primer sequences of target genes were described in Table 1.

**TABLE 1:** Sequences of primers used for real-time quantitative PCR.

Gene	Forward primer sequence (5′–3′)	Reverse primer sequence (5′–3′)
Gapdh	AGA​ACA​TCA​TCC​CTG​CAT​CC	TTG​TCA​TTG​AGA​GCA​ATG​CC
Fasn	GCC​ATG​CCC​AGA​GGG​TGG​TT	AGG​GTC​GAC​CTG​GTC​CTC​A
Acaca	TGG​AGC​TAA​ACC​AGC​ACT​CC	GCC​AAA​CCA​TCC​TGT​AAG​CC
Cpt1a	ATC​GTG​GTG​GTG​GGT​GTG​ATA​T	ACG​CCA​CTC​ACG​ATG​TTC​TTC
Acox	TGT​CAT​TCC​TAC​CAA​CTG​TC	CCA​TCT​TCT​CAA​CTA​ACA​CTC
CD36	GTG​CAA​AAC​CCA​GAT​GAC​GT	TCC​AAC​AGA​CAG​TGA​AGG​CT
Fabp1	GCA​GAG​CCA​GGA​GAA​CTT​TGA​G	TTT​GAT​TTT​CTT​CCC​TTC​ATG​CA
Mttp	AAC​TCC​TAC​GAG​CCC​TCC​TT	AGT​CCT​CCC​AGG​ATC​AGC​TT
Apob	TCA​CCA​TTT​GCC​CTC​AAC​CTA​A	GAA​GGC​TCT​TTG​GAA​GTG​TAA​AC
*Ppara*	AGA​GCC​CCA​TCT​GTC​CTC​TC	ACT​GGT​AGT​CTG​CAA​AAC​CAA​A

### 2.7 Western blot

Total proteins in the liver samples were extracted by the lysis buffer containing 4% protease inhibitor cocktail (Cat#4693132001, Roche, 25×) and 10% phosphatase inhibitor cocktail (Cat#4906837001, Roche, 10×) (Zhang et al., 2020b). The expression levels of the corresponding proteins in the liver samples were determined by Western blot according to the standard processes (Zhang et al., 2021a). The antibodies used in this study were listed in Table 2.

**TABLE 2:** The antibodies.

Antibodies	Catalog numbers	Suppliers
Anti-rabbit IgG HRP-linked antibody	#S0001	Affinity Biosciences
Anti-mouse IgG HRP-linked antibody	#S0002	Affinity Biosciences
TNF-α antibody	#AF7014	Affinity Biosciences
Collagen I Antibody	#AF7001	Affinity Biosciences
Collagen III Antibody	#AF0136	Affinity Biosciences
ASC	#DF6304	Affinity Biosciences
TLR4 antibody	#sc-293072	Santa Cruz Biotechnology
Caspase-3 antibody	#sc56053	Santa Cruz Biotechnology
Caspase-11 antibody	#sc-374615	Santa Cruz Biotechnology
Caspase-8 antibody	#9746S	Cell Signaling Technology
p-NF-κB p65 antibody	#3033S	Cell Signaling Technology
NF-κB p65 antibody	#8242S	Cell Signaling Technology
NLRP3	#15101S	Cell Signaling Technology
Phospho-SAPK/JNK antibody	#9255S	Cell Signaling Technology
SAPK/JNK Antibody	#9252S	Cell Signaling Technology
p38 antibody	#8690S	Cell Signaling Technology
p-p38 antibody	#4511S	Cell Signaling Technology
MAPK (Erk1/2) antibody	#4695S	Cell Signaling Technology
Rabbit Anti-phospho-ERK1/2 (Thr202 + Tyr204) antibody	bs-3016R	Bioss
IL-18 antibody	#D046-3	Medical and Biological Laboratories Co., Ltd.
GAPDH antibody	#MB001	Bioworld Technology
3-nitrotyrosine (3-NT) antibody	#ab110282	Abcam
GSDMD antibody	#ab219800	Abcam
GSDME antibody	#ab215191	Abcam
Caspase-1 antibody	#ab1872	Abcam

### 2.8 Immunofluorescence staining

3-NT immunofluorescence staining was performed on frozen sections of the liver samples as follows: (1) Wash frozen sections with PBS for 30 min, with TBS for 10 min, and with TBST for 20 min (2) Seal with goat serum blocking solution (Cat#ZLI-9056, ZSGB Bio, Beijing, China) at room temperature for 2 h (3) Incubate overnight with 3-NT antibody (see Table 1) at 4°C. (4) Wash with TBS for 10 min, with TBST for 20 min (5) Incubate with Goat Anti-Mouse IgG (H+L) Fluor 594-conjugated secondary antibody (Cat#S0005, Affinity Biosciences, Beijing, China) under dark conditions for 2 h (6) Wash with TBS for 10 min, with TBST for 20 min (7) Finally, the fluorescence intensity of 3-NT was observed under a fluorescence microscope after sealing the sections with Mounting Medium, antifading (with DAPI) (Cat#S2110, Solarbio, Beijing, China).

### 2.9 Statistical analysis

The statistical analyses were performed by one-way analysis of variance (ANOVA) followed by Bonferroni’s *post hoc* analysis for data with normal distribution (by Shapiro-Wilk test) and satisfying homogeneity of variance (by Brown-Forsythe test), performed by Brown-Forsythe and Welch ANOVA tests followed by Dunnett T3 *post hoc* analysis for data with normal distribution and heteroscedasticity, and performed by Kruskal-Wallis test followed by Dunn’s *post hoc* analysis for data with skewed distributions (by Shapiro-Wilk test). All data were expressed as mean ± SD or median ± interquartile range, a value of P < 0.05 was considered as significantly different. All histograms were performed using GraphPad Prism 10.1.2 (GraphPad Software Inc., San Diego, CA, United States).

## 3 Results

### 3.1 H_2_ improved hepatic steatosis in mice fed with a MCD diet

**FIGURE 2:**
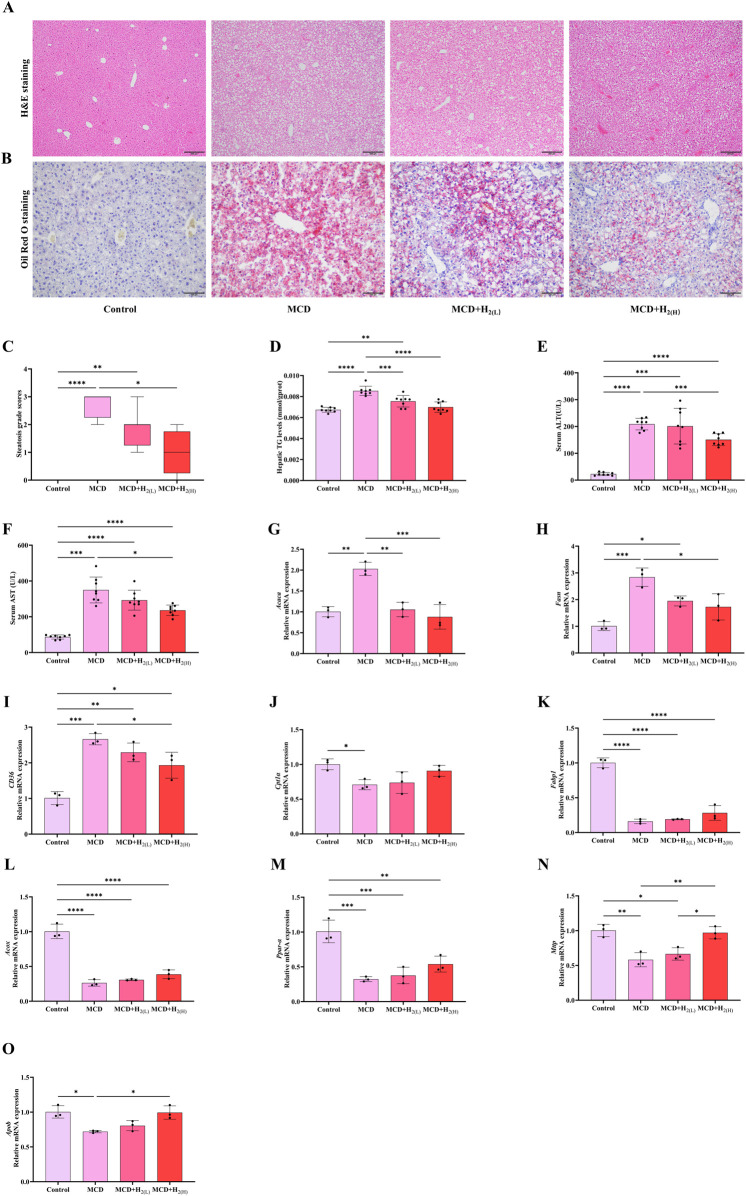
H_2_ therapy improved hepatic steatosis in mice fed with a MCD diet. **(A)** Liver H&E and **(B)** Oil Red O staining showed that both low and high doses of H_2_ attenuated MCD-induced hepatic steatosis. **(C)** Steatosis grade score, n = 8 mice in each group. **(D)** The levels of TG in the liver tissues, **(E)** Serum ALT levels, **(F)** Serum AST levels, n = 8 mice in each group. **(G)** The relative mRNA levels (ratios to *Gapdh*) of *Acaca*, **(H)** *Fasn*, **(I)** *CD36*, **(J)** *Cpt1α*, **(K)** *Fabp1*, **(L)** *Acox*, **(M)** *Ppar-α*, **(N)** *Mttp*, **(O)** *Apob*, n = 3 mice in each group. The data of steatosis grade score are expressed as median ± interquartile range, other results are expressed as means ± SD. ^*^p < 0.05, ^**^p < 0.01, ^***^p < 0.001, ^****^p < 0.0001.

The basic characteristics of MASLD is hepatic steatosis (Rinella et al., 2023; Hagström et al., 2024). Therefore, we first investigated that whether supplementing with H_2_ through intraperitoneal injection has a protective effect on lipid deposition in the liver of mice fed with a MCD diet. Macrovesicular steatosis indicated by H&E staining (Figure 2A) and lipid droplets indicated by Oil red O staining (Figure 2B) were obviously visible in MCD group. The steatosis grade scores (Figure 2C), and hepatic TG levels (Figure 2D) were higher in MCD group than Control group. The increased serum levels of ALT (Figure 2E) and AST (Figure 2F) pointed to hepatocellular injury in MCD group. All these indicators reflecting hepatic steatosis and liver injury were improved by high dose H_2_ therapy. Hepatic steatosis is a consequence of lipid acquisition exceeding lipid disposal, for example, fatty acid uptake and *de novo* lipogenesis surpassing lipid export and fatty acid oxidation (Ipsen et al., 2018). Here, we found the hepatic mRNA involved in *de novo* lipogenesis, such as acetyl-Coenzyme A carboxylase (*Acaca*) and fatty acid synthetase (*Fasn*), and fatty acid uptake gene *CD36* were increased by feeding a MCD diet for 3 weeks (Figures 2G-I); in contrast, the genes involved in fatty acid oxidation, such as carnitine palmitoyl transferase 1 α (*Cpt1α*) and fatty acid binding protein 1 (*Fabp1*), Acyl-CoA oxidase (*Acox*, the rate-limiting enzyme in peroxisomal β-oxidation of fatty acids), and peroxisome proliferator-activated receptor-α (*PPAR-α*) (Figures 2J-M), the lipid exporting genes such as microparticle triglyceride transfer protein (*Mttp*) and apolipoprotein B (*Apob*) were decreased by feeding a MCD diet for 3 weeks (Figures 2N,O). Among these, *Acaca*, *Fasn*, and *CD36* were decreased (Figures 2G-I), while *Mttp* and *Apob* were increased by high dose H_2_ therapy (Figures 2N,O). Therefore, these data indicated that H_2_ can improve hepatic steatosis in mice fed with a MCD diet probably via inhibiting *de novo* lipogenesis and fatty acid uptake, while increasing lipid export.

### 3.2 H_2_ improved liver fibrosis in mice fed with a MCD diet

**FIGURE 3:**
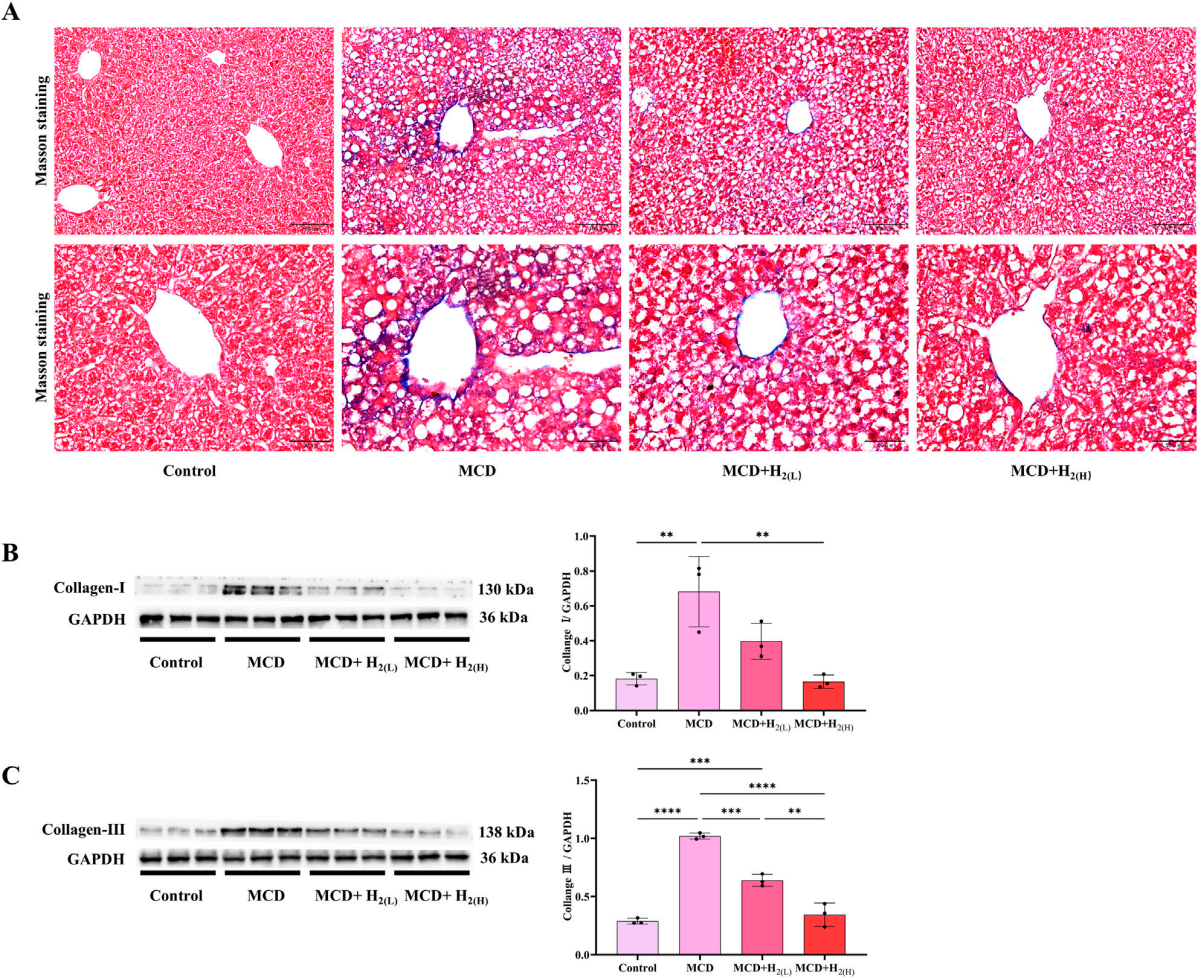
The effect of H_2_ on liver fibrosis in mice fed with a MCD diet. **(A)** The Masson’s trichrome staining showed that H_2_ alleviated mild liver fibrosis induced by MCD. **(B)** Western blotting images of hepatic Collagen-Ⅰ and GAPDH, and quantification of Collagen-Ⅰ/GAPDH ratio, **(C)** Western blotting images of hepatic Collagen-Ⅲ and GAPDH, and quantification of Collagen-Ⅲ/GAPDH ratio, n = 3 mice in each group. Results are expressed as means ± SD. ^**^p < 0.01, ^***^p < 0.001, ^****^p < 0.0001.

MASLD patients have the potential for progressively developing into liver fibrosis (Chalasani et al., 2017; Younossi et al., 2023). Our Masson staining indicated that MCD diet feeding induced liver fibrosis in mice, and the hepatic protein levels of fibrosis markers Collagen-Ⅰ and Collagen-Ⅲ, were increased in mice fed with a MCD diet, these conditions were all improved by H_2_ therapy (Figure 3). Therefore, these data indicated that H_2_ also has the potential to improve liver fibrosis in mice fed with a MCD diet.

### 3.3 H_2_ alleviated oxidative stress in the liver of mice fed with a MCD diet

**FIGURE 4:**
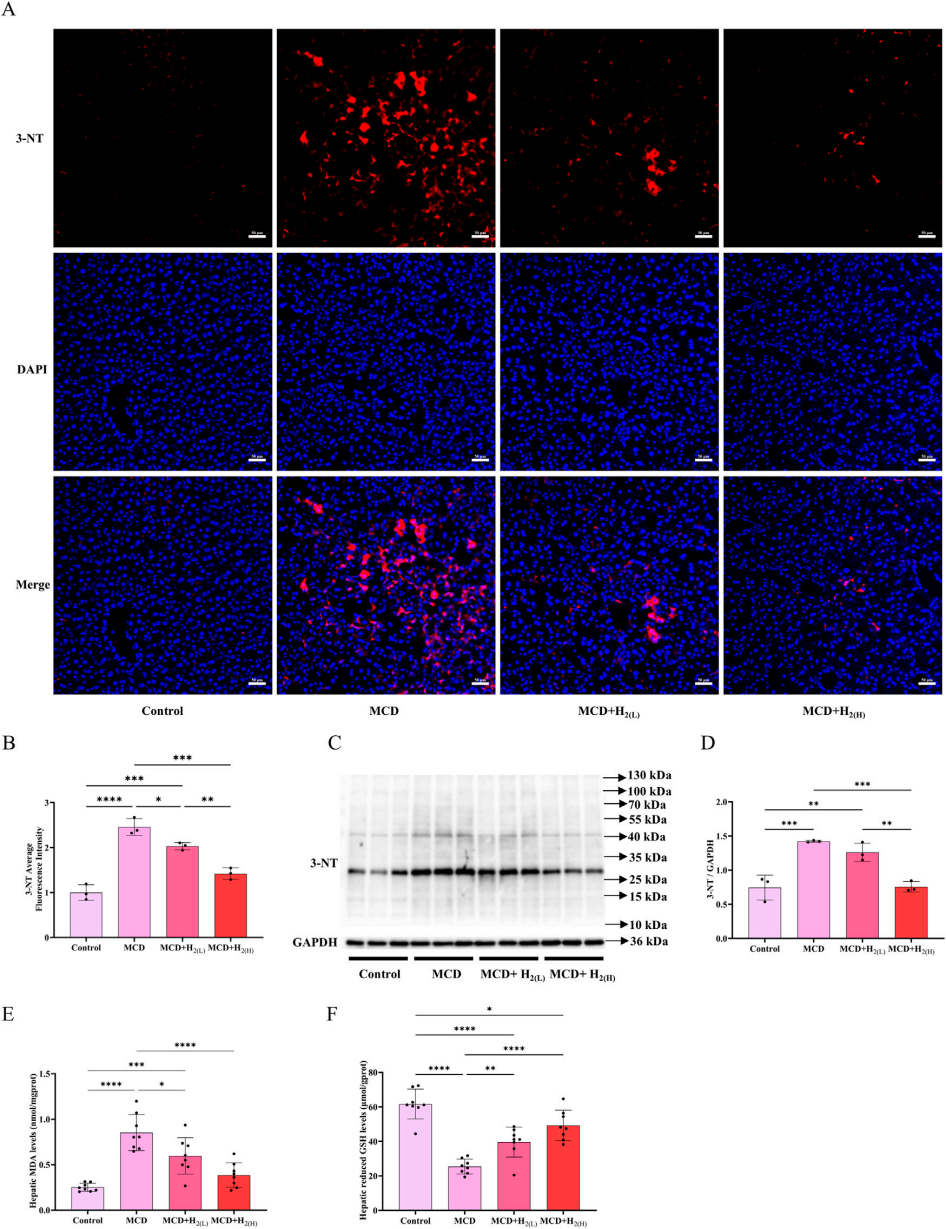
H_2_ alleviated oxidative stress in the liver of mice fed with a MCD diet. **(A)** Immunofluorescence of 3-NT, **(B)** 3-NT average fluorescence intensity, **(C)** Western blotting images of hepatic 3-NT and GAPDH and **(D)** quantification of 3-NT/GAPDH ratio, n = 3 mice in each group. **(E)** Hepatic MDA and **(F)** the reduced GSH levels, n = 8 mice in each group. Results are expressed as means ± SD. ^*^p < 0.05, ^**^p < 0.01, ^***^p < 0.001, ^****^p < 0.0001.

Hepatic oxidative stress is a key feature and contributor of MASLD (Li et al., 2024). To investigate the effects of intraperitoneal injection of H_2_ on oxidative damage in the liver of mice fed with a MCD diet, hepatic 3-nitrotyrosine (3-NT), an indicator of oxidative stress, were examined by immunofluorescence and Western blot. Compared with Control group, 3-NT levels in the liver were elevated in MCD group, and it was decreased by H_2_ therapy (Figures 4A–D). Malondialdehyde (MDA) is an aldehyde formed as secondary products during lipid peroxidation, in contrast, glutathione (GSH) is a principal intracellular antioxidant buffer (Ayala et al., 2014; Wrotek et al., 2020). We further evaluated MDA and GSH levels in the liver. Hepatic MDA levels were increased, while hepatic reduced GSH levels were decreased in MCD group when compared with Control group, in contrast, H_2_ therapy reversed this redox imbalance (Figures 4E,F). Therefore, H_2_ therapy alleviated oxidative stress in the liver of mice fed with a MCD diet.

### 3.4 H_2_ suppressed pyroptosis in the liver of mice fed with a MCD diet

**FIGURE 5:**
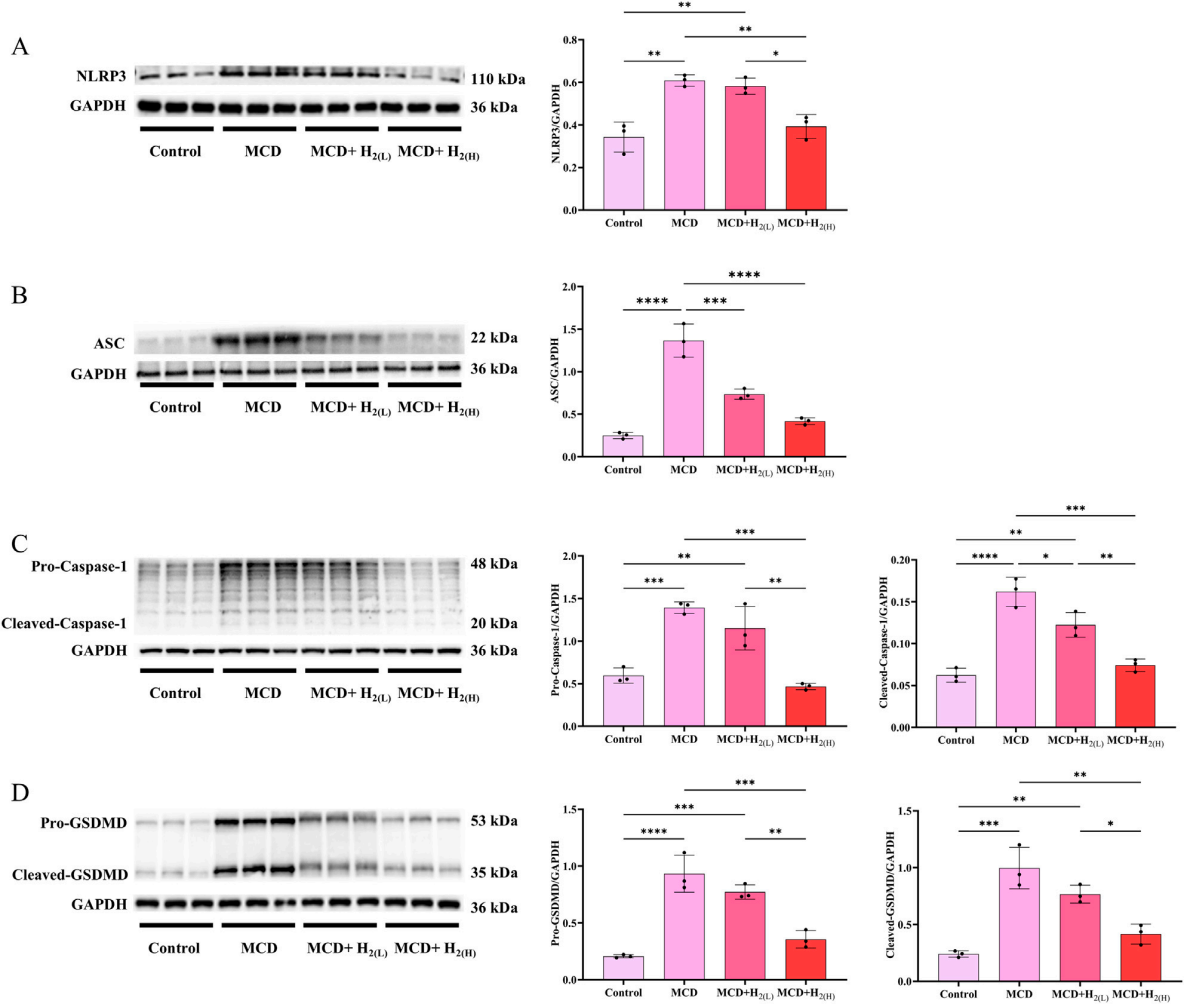
H_2_ therapy inhibited hepatic NLRP3 inflammasome activation in mice fed with a MCD diet. **(A)** Western blotting images of hepatic NLRP3 and GAPDH, and quantification of NLRP3/GAPDH ratio; **(B)** Western blotting images of hepatic ASC and GAPDH, and quantification of ASC/GAPDH ratio; **(C)** Western blotting images of hepatic pro-Caspase-1, cleaved-Caspase-1 and GAPDH, and quantifications of pro-Caspase-1/GAPDH and cleaved-Caspase-1/GAPDH ratios; **(D)** Western blotting images of pro-GSDMD, cleaved-GSDMD and GAPDH in the liver, and quantifications of pro-GSDMD/GAPDH and cleaved-GSDMD/GAPDH ratios; n = 3 mice in each group. Results are expressed as means ± SD. ^*^p < 0.05, ^**^p < 0.01, ^***^p < 0.001, ^****^p < 0.0001.

**FIGURE 6:**
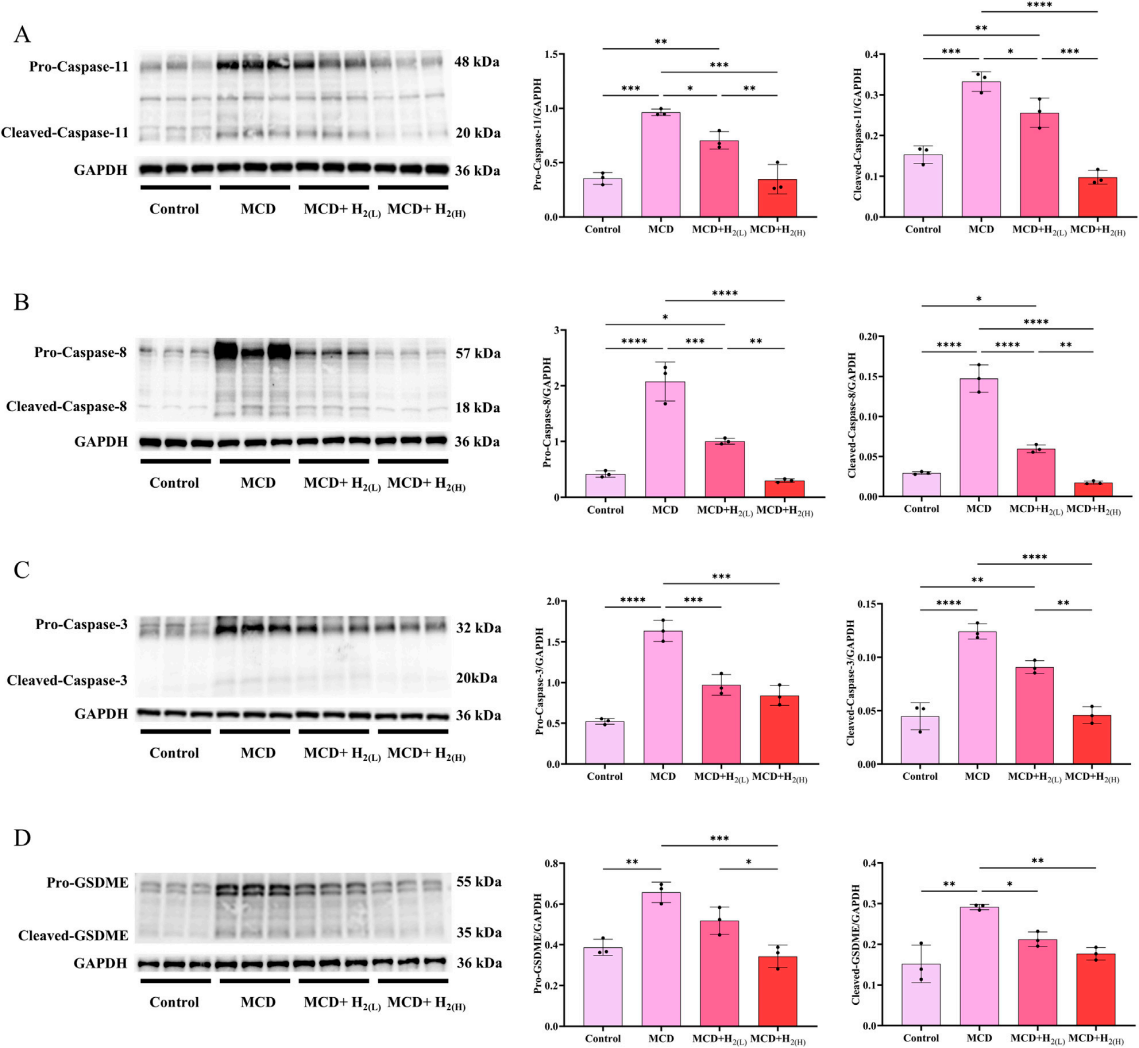
H_2_ therapy inhibited the non-classical pyroptosis signaling in the liver of mice fed with a MCD diet. **(A)** Western blotting images of pro-Caspase-11, cleaved-Caspase-11 and GAPDH in the liver, and quantifications of pro-Caspase-11/GAPDH and cleaved-Caspase-11/GAPDH ratios; **(B)** Western blotting images of pro-Caspase-8, cleaved-Caspase-8 and GAPDH in the liver, and quantifications of pro-Caspase-8/GAPDH and cleaved-Caspase-8/GAPDH ratios; **(C)** Western blotting images of pro-Caspase-3, cleaved-Caspase-3 and GAPDH in the liver, and quantifications of pro-Caspase-3/GAPDH and cleaved-Caspase-3/GAPDH ratios; **(D)** Western blotting images of pro-GSDME, cleaved-GSDME and GAPDH in the liver, and quantifications of pro-GSDME/GAPDH and cleaved-GSDME/GAPDH ratios; n = 3 mice in each group. Results are expressed as means ± SD. ^*^p < 0.05, ^**^p < 0.01, ^***^p < 0.001, ^****^p < 0.0001.

In human and murine models of MASH, increased hepatocyte death, such as apoptosis and pyroptosis, is a critical mechanism contributing to inflammation and fibrogenesis (Hatting et al., 2013; Xu et al., 2018; Gaul et al., 2021). The canonical proptosis, a lytic form of cell death, can be elicited by Caspase-1 (which is activated after the binding of the ligands to the inflammasome-forming pattern recognition receptors (PRRs), such as NLRP3 (Shi et al., 2015)) to cleave the pyroptosis executioner, gasdermin D (GSDMD) (Shi et al., 2017). The gasdermin-N domains of GSDMD can bind the membrane lipids, phosphoinositides and cardiolipin, and exhibit membrane-disrupting cytotoxicity in mammalian cells, and thus triggering pyroptosis (Ding et al., 2016). In order to investigate the effect of H_2_ on NLRP3 inflammasome activation and the subsequent pyroptosis in the liver of mice fed with a MCD diet, we examined NLRP3, ASC, Caspase-1 and GSDMD protein levels in the liver. Our results showed that MCD diet feeding increased the protein levels of NLRP3 and ASC, and the full length and cleaved forms of Caspase-1 and GSDMD in the liver, in contrast, these upregulation were decreased by H_2_ therapy (Figure 5). In addition to Caspase-1-GSDMD pathway, the non-canonical pathway can also induce pyroptosis, as the cleavage of GSDMD by Caspaes-11/8 and the cleavage of GSDME specifically by Caspase-3 (Kayagaki et al., 2015; Wang et al., 2017; Sarhan et al., 2018). Here, we showed that compared with Control group, the full length and cleaved forms of Caspase-11, Caspase-8, Caspase-3, and GSDME were increased, while H_2_ downregulated both the expression and maturation of these pyroptosis signaling proteins (Figure 6). Therefore, H_2_ inhibited pyroptosis in the liver of mice fed with a MCD diet.

### 3.5 H_2_ therapy reduced inflammatory cytokines expression in the liver of mice fed with a MCD diet

**FIGURE 7:**
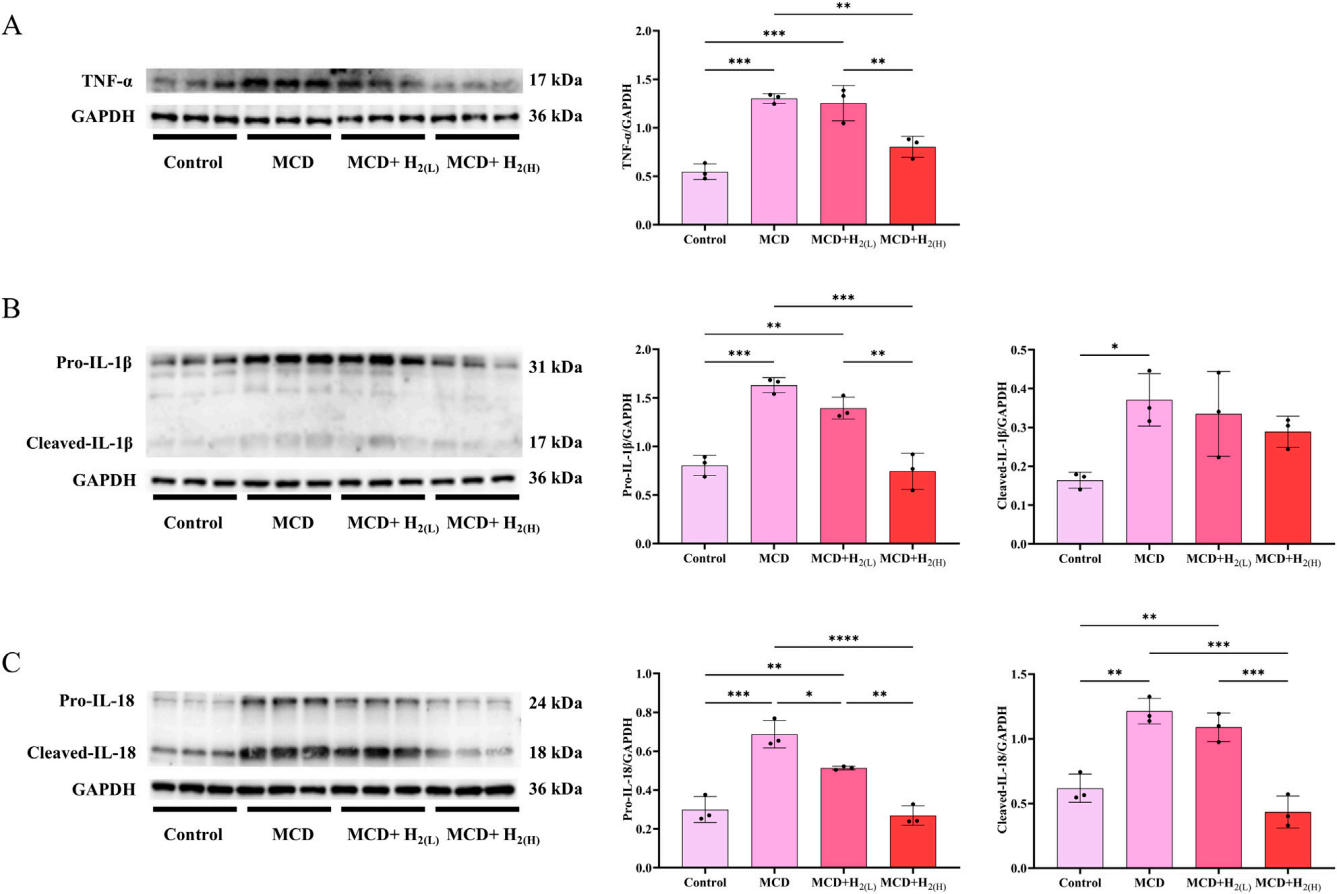
H_2_ suppressed hepatic pro-inflammatory cytokines expression in mice fed with a MCD diet. **(A)** Western blotting images of TNF-α and GAPDH in the liver, and quantifications of TNF-α/GAPDH ratio, **(B)** Western blotting images of pro-IL-1β, cleaved- IL-1β and GAPDH in the liver, and quantifications of pro-IL-1β/GAPDH and cleaved- IL-1β/GAPDH ratios, **(C)** Western blotting images of pro-IL-18, cleaved- IL-18 and GAPDH in the liver, and quantifications of pro-IL-18/GAPDH and cleaved-IL-18/GAPDH ratios, n = 3 mice in each group. Results are expressed as means ± SD. ^*^p < 0.05, ^**^p < 0.01, ^***^p < 0.001, ^****^p < 0.0001.

The activated Caspase-1 can process the cleavage of GSDMD and IL-1β, and the cleavage of GSDMD is required for pyroptosis and the release of matured IL-1β (He et al., 2015; Shi et al., 2015). It is well-known that the increased inflammatory cytokines were essential for the pathogenesis of MASLD. Therefore, we examined hepatic protein levels of inflammatory cytokines to explore the effects of intraperitoneal injection of H_2_ on inflammation in mice with MASLD. Our results showed that compared with Control group, the hepatic levels of TNF-α, the full length and cleaved forms of IL-1β and IL-18 were all increased in MCD group, and high dose H_2_ therapy reversed the upregulation of these inflammatory cytokines in the liver (Figure 7). Therefore, H_2_ therapy inhibited inflammatory cytokines expression in the liver of mice fed with a MCD diet.

### 3.6 H_2_ inhibited the overactivation of TLR4 innate immune signaling in the liver of mice fed with a MCD diet

**FIGURE 8:**
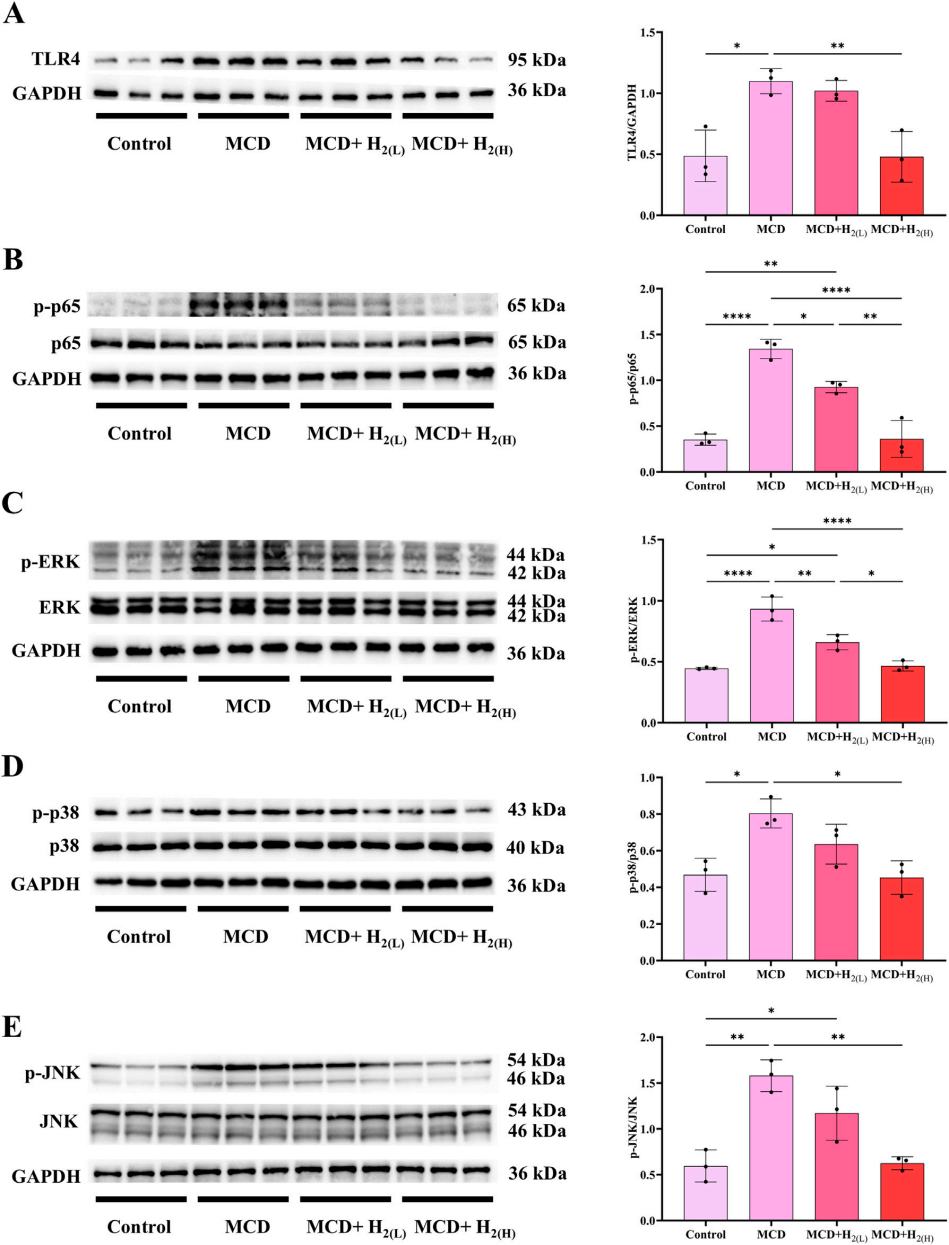
H_2_ suppressed hepatic TLR4-NFκB and MAPK innate immune signaling in mice fed with a MCD diet. **(A)** Western blotting images of TLR4 and GAPDH in the liver, and quantifications of TLR4/GAPDH ratio; **(B)** Western blotting images of p-p65, (total) p65 and GAPDH in the liver, and quantifications of p-p65/(total) p65 ratio; **(C)** Western blotting images of p-ERK, (total) ERK and GAPDH in the liver, and quantifications of p-ERK/(total) ERK ratio; **(D)** Western blotting images of p-p38, (total) p38 and GAPDH in the liver, and quantifications of p-p38/(total) p38 ratio; **(E)** Western blotting images of p-JNK, (total) JNK and GAPDH in the liver, and quantifications of p-JNK/(total) JNK ratio; n = 3 mice in each group. Results are expressed as means ± SD. ^*^p < 0.05, ^**^p < 0.01, ^***^p < 0.001, ^****^p < 0.0001.

The expression of hepatic inflammatory cytokines were induced by the activation of pattern recognition receptors (PRRs, such as Toll-like receptor 4 (TLR4)) after recognizing the upregulated pathogen-associated molecular patterns (PAMPs, such as LPS) or damage associated molecular patterns (DAMPs) during the progression of MASLD (Carpino et al., 2020). Here, the expression of TLR4, and the phosphorylation of its downstream signaling proteins, including nuclear factor-κB (NF-κB), ERK1/2, p38 MAPK, and JNK, were all increased in the liver of mice fed with a MCD diet (Figure 8). In contrast, this overactivated TLR4 innate immune signaling was suppressed by intraperitoneal injection of high doses H_2_ (Figure 8).

### 3.7 H_2_-rich medium improved sodium oleate-induced HepG2 cells steatosis by inhibiting inflammatory cytokines expression and pyroptosis

**FIGURE 9:**
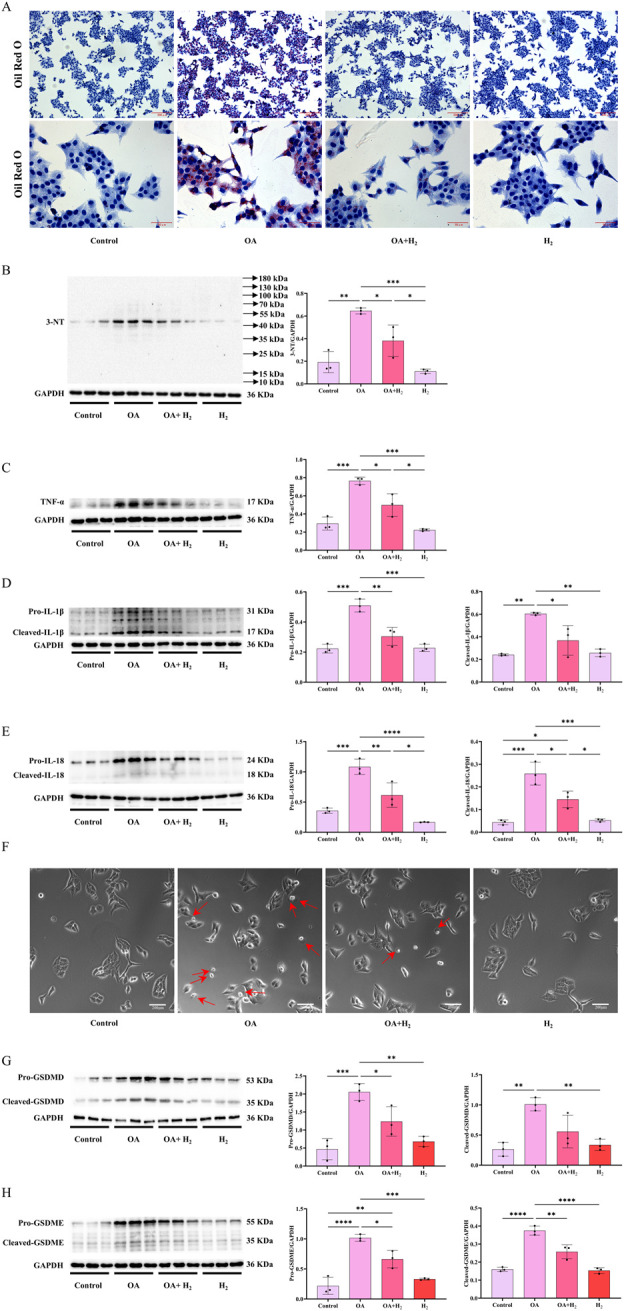
H_2_-rich medium improved OA-induced steatosis in HepG2 cells by inhibiting inflammatory cytokines expression and pyroptosis. **(A)** The Oil Red O staining of HepG2 cells. **(B)** Western blotting images of 3-NT and GAPDH in HepG2 cells, and quantifications of 3-NT/GAPDH ratio. **(C)** Western blotting images of TNFα and GAPDH in HepG2 cells, and quantifications of TNFα/GAPDH ratio. **(D)** Western blotting images of IL-1β and GAPDH in HepG2 cells, and quantifications of pro-IL-1β/GAPDH and cleaved-IL-1β/GAPDH ratios. **(E)** Western blotting images of IL-18 and GAPDH in HepG2 cells, and quantifications of pro-IL-18/GAPDH and cleaved-IL-18/GAPDH ratios. **(F)** The images of pyroptosis morphology of HepG2 cells observed under the bright field. **(G)** Western blotting images of pro-GSDMD, cleaved-GSDMD and GAPDH in the liver, and quantifications of pro-GSDMD/GAPDH and cleaved-GSDMD/GAPDH ratios. **(H)** Western blotting images of pro-GSDME, cleaved-GSDME and GAPDH in HepG2 cells, and quantifications of pro-GSDME/GAPDH and cleaved-GSDME/GAPDH ratios. Results are expressed as means ± SD. ^*^p < 0.05, ^**^p < 0.01, ^***^p < 0.001, ^****^p < 0.0001.

The hepatic protection of H_2_ on steatosis was further confirmed in sodium oleate (OA)-induced HepG2 cells steatosis model as indicated by Oil red O staining (Figure 9A). OA increased the expression of 3-NT (Figure 9B), TNFα (Figure 9C), IL1-β (Figure 9D) and IL-18 (Figure 9E) in HepG2 cells, these were all suppressed by H_2_-rich medium. Moreover, OA increased the numbers of cells with the pyroptosis morphology as cell swelling with large bubbles (Figure 9F), increased the levels of full-length and cleaved forms of GSDMD (Figure 9G) and GSDME (Figure 9H), these indicated that OA elicited pyroptosis in HepG2 cells, which were all inhibited by H_2_-rich medium treatment. Therefore, H_2_-rich medium inhibited the expression of inflammatory cytokines, and targeted GSDMD and GSDME to alleviate OA-induced steatosis in HepG2 cells.

## 4 Discussion

MASLD is the leading chronic liver disease worldwide. The spectrum of MASLD ranges from simple steatosis, through MASH, to fibrosis, and ultimately cirrhosis and HCC. Until March 2024, the US Food and Drug Administration (FDA) approved the launch of the first (and only) characteristic drug, resmetirom, for the treatment of fibrosis in MASH (Keam, 2024). Therefore, it is still urgent to find other effective treatments for MASH. H_2_ is a gas molecule with anti-inflammation, anti-oxidation, and anti-apoptosis effects. Since Ikuroh Ohsawa first discovered that inhaling 2% H_2_ can improve cerebral I/R injury in rats, the therapeutic effect of H_2_ has attracted more attention (Ohsawa et al., 2007). So far, H_2_ has been reported to have therapeutic effects in various liver diseases, such as alcoholic liver injury (Zhang et al., 2021a; Xu et al., 2024). Here, we first reported that intraperitoneal injection of H_2_, as a novel strategy for supplementing exogenous H_2_, can treat MCD diet-induced MASLD in mice. Future research should add a positive control group, using Resmetirom, to evaluate the efficacy of H_2_.

Methionine or choline can stimulate the synthesis of phosphatidylcholine (the principal phospholipid comprising the outer coat of very-low-density lipoprotein (VLDL) particles), which is required for the secretion of VLDL and its deficiency induces lipid accumulation in the liver (Rizki et al., 2006). Our data showed that MCD diet feeding induced obvious hepatic steatosis in mice as indicated by H&E and oil red O staining, and the increased hepatic TG levels. MCD diet promotes hepatic lipid accumulation by more than one mechanism (Rinella et al., 2008). The four key events as *de novo* lipogenesis, fatty acid uptake, lipid export and fatty acid oxidation, are all involved in the pathogenesis of MASLD in animals induced by feeding a MCD diet, however, the degree of MASLD is related to the days of feeding, the differences in MCD dietary components, and the animal strains (Kirsch et al., 2003; Rizki et al., 2006; Gyamfi et al., 2008; Rinella et al., 2008; Pickens et al., 2009; Wu et al., 2010; Machado et al., 2015; Pierce et al., 2015; Xu et al., 2018; Yu et al., 2019). Here, we found that the genes involved in fatty acid uptake (*CD36*) and *de novo* lipogenesis (*Acaca* and *Fasn*) were increased and the genes involved in lipid export (*Mttp* and *Apob*) and fatty acid oxidation (Cpt1α, *Fabp1*, *Acox*, and *PPAR-α*) were decreased in the liver of male C57BL/6 mice fed with a MCD diet for 3 weeks. H_2_ treatment decreased the mRNA levels of genes involved in *de novo* lipogenesis and fatty acid uptake, while increased the mRNA levels of genes involved in lipid export. In order to provide a precise answer on the effect of H_2_ on hepatic lipid metabolism, we should further detect the protein levels and activities of these genes, and levels of related metabolites. H_2_ also improve liver fibrosis and decreased hepatic Collagen-Ⅰ and Collagen-Ⅲ protein levels induced by MCD diet feeding. Therefore, H_2_ has a therapeutic effect on MASLD in mice caused by feeding a MCD diet.

The pathophysiology underlying MASLD is complex and incompletely understood. In 1998, Day and James proposed two hits hypothesis, that the first hit is steatosis, and the second hit is oxidative stress (Day and James, 1998). In 2010, Tilg and Moschen further proposed multiple parallel hits hypothesis, many parallel hits, such as lipotoxicity, insulin resistance, oxidative stress, and the overactivation of both innate and adaptive immunity, contribute to the development of MASLD or MASH (Tilg and Moschen, 2010; Targher et al., 2024). Among these, oxidative stress is as a central mechanism driving MASLD, it results from an imbalance between the production and elimination of ROS (Hu et al., 2025). Here, oxidative stress indicators including 3-NT and MDA increased, and the antioxidant GSH in the liver of mice fed with a MCD diet decreased, these indicated that MCD diet feeding disturbed hepatic redox homeostasis, which was improved by H_2_ treatment. It is known that H_2_ selectively reduced the hydroxyl radical, the most cytotoxic of ROS, and effectively protected cells (Ohsawa et al., 2007). H_2_ can also reduce the expression of NADPH oxidase subunit p67 (phox) expression (Zhang et al., 2016). However, the detail mechanism by which H_2_ improves redox homeostasis in MASLD still requires further exploration.

**FIGURE 10:**
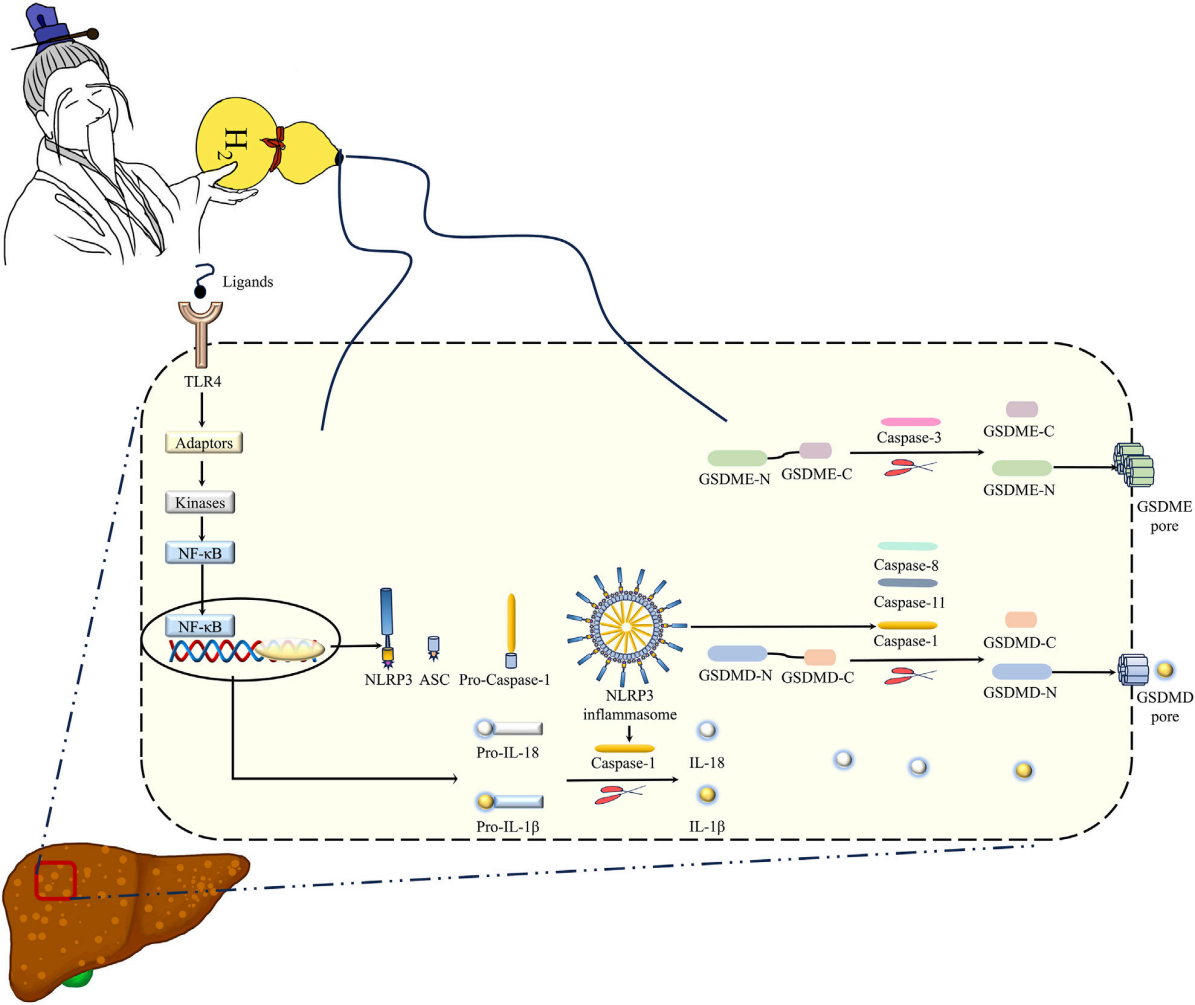
Hydrogen gas (H_2_) is the immortal energy that subdues demons and monsters. H_2_ is like the immortal qi in the treasure gourd of the immortal grandfather in Chinese mythology, which can subdue demons and eliminate evil spirits. Intraperitoneal injection of H_2_, as a novel method for H_2_ delivery, can alleviate MCD diet-induced MASLD in mice via inhibiting hepatic oxidative stress, TLR4-mediated innate immune signaling, and GSDMD- and GSDME-mediated pyroptosis. Therefore, H_2_ therapy may act as a novel therapeutic strategy for MASLD in animals, which is worth further investigation.

Pyroptosis is a key mechanism of MASLD (Xu et al., 2018; Gaul et al., 2021). Pyroptosis is executed by the pore-forming protein GSDMD, which is always activated/cleaved by Caspase-1, murine Caspase-11 and its human orthologs Caspase-4 and Caspase-5, and Caspase-8 (Shi et al., 2017; Orning et al., 2018; Sarhan et al., 2018; Rathinam et al., 2019). The activated canonical inflammasomes, such as NLRP3 inflammasome, by the ligands can activate Caspase-1; Caspase-4, Caspase-5, and Caspase-11 can directly recognize bacterial LPS; both of which trigger pyroptosis by cleaved GSDMD (Shi et al., 2015). NLRP3 inflammasome includes three major components: NLRP3, Caspase-1, and ASC (apoptosis-associated speck-like protein containing a caspase recruitment domain), which acts as a bridge connecting NLRP3 and Caspase-1 (Fu and Wu, 2023). The protein levels of NLRP3, ASC, full-length and the cleaved forms of Caspase-1 and GSDMD in the liver were increased by feeding a MCD diet, and these upregulation were decreased by intraperitoneal injection of H_2_. These indicated that H_2_ can inhibit NLRP3 inflammasome-mediated canonical pyroptosis signaling in the liver of MASLD mice. This canonical pyroptosis signaling was also inhibited by H_2_ inhalation in myocardial infarction rat model and in myocardial I/R injury rat model (Nie et al., 2021a; Nie et al., 2021b). The non-canonical pyroptosis signaling, such as Caspase-11 and Caspase-8 to cleave GSDMD, and Caspase-3 to cleave GSDME (Kayagaki et al., 2015; Wang et al., 2017; Sarhan et al., 2018), were increased in the liver of mice fed with a MCD diet. These non-canonical pyroptosis signaling were suppressed by H_2_ therapy. Recently, our group also showed that intraperitoneal injection of H_2_ can alleviate acute ethanol-induced hepatotoxicity in mice partially via inhibiting Caspase-11 and Caspase-8 to GSDMD, and Caspase-3 to GSDME non-canonical pyroptosis signaling in the liver (Xu et al., 2024). These indicate that H_2_ is an anti-pyroptosis gas molecule, and this is one of the key mechanisms of H_2_ on treating MASLD in mice (Figure 10).

In addition to cleaving GSDMD to trigger pyroptosis, the cleaved Caspase-1 can also induced the cleavage of IL-1β and IL-18, and GSDMD is not only an executor of pyroptosis, but is also required for IL-1β secretion (He et al., 2015; Shi et al., 2015). As mentioned above, inflammation is also one of the key pathogeneses of MASLD. MCD diet feeding increased hepatic levels of TNF-α, the full length and cleaved forms of IL-1β and IL-18, and these can be suppressed by H_2_. Like this, H_2_ also showed the anti-inflammation effect in the heart, brain, kidney, and the sex organs (Zhang et al., 2018; Zhang et al., 2020a; Zhang et al., 2021b). We, therefore, investigated the mechanism of downregulation of inflammatory cytokines by H_2_. It has been reported that LPS, which can be recognized by the PRRs such as TLR4, was increased in MASLD mice model and patients (Carpino et al., 2020). TLR4 can elicit IKK phosphorylation to induce NF-κB-mediated inflammatory cytokines expression, or elicit MAPK (ERK1/2, p38 MAPK and JNK) phosphorylation to induce AP-1-mediated inflammatory cytokines expression (Zhang et al., 2017a; Zhang and Li, 2017; Zhang et al., 2017b). The expression of TLR4, the phosphorylation of NF-κB, ERK1/2, p38 MAPK and JNK were increased in the liver of mice fed with a MCD diet. H_2_ can reverse the overactivation of TLR4-mediated innate immune signaling in the liver (Figure 10). Our previously study also showed that the activation of NF-κB and MAPK signaling were suppressed by H_2_ in animal models of alcoholic liver disease and septic cardiomyopathy. However, it is unclear whether H_2_ can directly inhibits the phosphorylation of these molecules or suppressed their upstream molecules, or indirectly activates the negative molecules of innate immunity.

## Data Availability

The original contributions presented in the study are included in the article/supplementary material, further inquiries can be directed to the corresponding authors.
